# A Solution to the Cerebral Perfusion Pressure Transducer Placement Conundrum in Neurointensive Care? The Dual Transducer

**DOI:** 10.1007/s12028-023-01829-8

**Published:** 2023-09-11

**Authors:** Era Mikkonen, Jonas Blixt, Ari Ercole, Peter Alpkvist, Robert Sköldbring, Bo-Michael Bellander, Eddie Weitzberg, David W. Nelson

**Affiliations:** 1 Department of Perioperative Medicine and Intensive Care, Karolinska University Hospital, Stockholm, Sweden; 2 Section of Perioperative Medicine and Intensive Care, Department of Physiology and Pharmacology, Karolinska Institutet, Stockholm, Sweden; 3 Division of Anaesthesia, University of Cambridge, Cambridge, UK; 4 Section for Neurosurgery, Department of Clinical Neuroscience, Karolinska Institutet, Stockholm, Sweden

**Keywords:** Brain injury, Patient monitoring, Cerebrovascular monitoring, Intracranial pressure, Critical care, Transducers, Pressure, 1. Background

## Abstract

Intracranial pressure is routinely monitored in most intensive care units caring for patients with severe neurological insults and, together with continuous arterial blood pressure measurement, allows for monitoring of cerebral perfusion pressure (CPP). CPP is the driving pressure of blood flow to the brain and is used to guide therapy. However, there is considerable inconsistency in the literature regarding how CPP is technically measured and, more specifically, the appropriate placement of the arterial pressure transducer. Depending on patient positioning and where the arterial pressure transducer is placed, the mean arterial pressure used for CPP calculation can vary widely by up to 15 mm Hg, which is greater than the acceptable variation in target ranges used clinically. Physiologically, the arterial pressure transducer should be placed at the level of the foramen of Monro for CPP measurement, but it is commonly set at the level of the right atrium for systematic measurement. Mean arterial pressure measurement at the level of the right atrium can lead to overestimation and potentially critically low actual CPP levels when the head is elevated, and measurement at the level of the foramen of Monro will underestimate systemic pressures, increasing the risk of excessive and unnecessary use of vasopressors and fluid. At the Karolinska University Hospital neurointensive care unit, we have used a split dual-transducer system, measuring arterial pressure both at the level of the foramen of Monro and at the level of the right atrium from a single arterial source. In doing so, we work with constants and can monitor and target optimum arterial pressures to better secure perfusion to all organs, with potentially less risk of cerebral ischemia or overuse of vasopressors and fluids, which may affect outcome.

Since the introduction of intracranial pressure (ICP) measurements in our neurointensive care units, cerebral perfusion pressure (CPP) has been a cornerstone of neuromonitoring and treatment [1]. CPP is considered to be the driving pressure of blood through the brain and is commonly calculated as mean arterial pressure (MAP) − ICP. If cerebral autoregulation is intact, CPP is expected to have little effect on cerebral blood flow. However, because autoregulation is often impeded after brain injury [2–4] and fails when above or below the critical levels of autoregulation, CPP levels are expected to have a direct effect on local and global blood flow. Thus, adequate CPP-targeted management has consequently been proposed to potentially affect outcome [5]. However, given that most intensive care unit patients are treated with the head raised at approximately 30 degrees, variations in the positionings of the arterial pressure monitoring transducer can have serious implications for the resulting calculated CPP and systemic blood pressure measurements.

There is considerable inconsistency in the literature regarding how CPP is measured in detail and, more specifically, the placement and leveling point of the extraventricular drain and the arterial pressure transducer [6–9]. In a patient without cerebral injury, when only systemic blood pressures are of interest, it is recommended that the arterial pressure transducer be placed at the level of the right atrium, using the phlebostatic axis as anatomical landmark [10–12]. With this transducer placement, the literature suggests MAP values above 60–65 mm Hg to avoid hypoperfusion and injury to internal organs in critically ill adult patients [13]. However, in a patient with cerebral injury lying at a 30° head elevation, one is interested in what pressures perfuse the brain, and leveling the transducer at the phlebostatic axis will overestimate the MAP at the level of the foramen of Monro by approximately 10–13 mm Hg. These pressure differences ($\Delta P$) between MAP measured at the phlebostatic axis (MAP_phlebo_) and MAP measured at the tragus (MAP_tragus_) during head elevation have previously been documented and are in concordance with undisputed geometrical calculations and Bernoulli’s equation [6, 14, 15]:

1 $$P_{{{\text{phlebo}}}} + \rho gh_{{{\text{phlebo}}}} = P_{{{\text{tragus}}}} + \rho gh_{{{\text{tragus}}}} ,$$

where $P$ is the measured pressure, $h_{{{\text{phlebo}}/{\text{tragus}}}}$ is the height of the phlebostatic axis/tragus, $\rho$ is the density of the catheter saline, and $g \approx 9.81\;{\text{ms}}^{2}$ is the acceleration due to gravity (because we are considering a hydrostatic condition, we need not consider a kinetic/flow component). Therefore, the $\Delta P$ observed is simply the following:

2 $$\Delta P = \rho g\left( {h_{{{\text{tragus}}}} - h_{{{\text{phlebo}}}} } \right).$$

In a patient with a neurological insult, a low placement of the transducer will overread CPP by $\Delta P$, possibly increasing the risk of undertreatment, hypoperfusion, and cerebral ischemia. In contrast, if the transducer is placed at the level of the tragus, the calculated CPP will correspond more closely to the actual driving pressure in the brain, but systemic pressures will be underestimated by $\Delta P$ and, in reality, be much higher than the recorded pressures. This will usually not be a cause of concern, unless, for example, ICP is low and CPP targets are met, and adequate MAP_phlebo_ must be estimated from the MAP_tragus_. Because the MAP_tragus_ will be lower than the MAP_phlebo_, targeting MAP of 65 mm Hg with the transducer placed at the tragus can cause excessive use of vasopressors and fluids to meet blood pressure targets, potentially affecting outcome [16]. Hence, both transducer leveling options entail working with incorrect values from one of the organ’s systems of interest, being that of MAP_phlebo_ or MAP_tragus_, when there is head elevation. Moreover, the error is not consistent when head elevation is varied during patient care, with a risk of unintended and potentially harmful hypoperfusion or hyperperfusion of organs. Further, the variability in measurement results depending on transducer leveling can, together with other sources of inaccuracies, contribute to considerable confusion concerning optimal perfusion pressure targets and a lack of reproducibility of studies, as the optimal ranges discussed and studied are close to that of the differences seen in alternative measurement methods [9, 17].

The literature on CPP targets has not been consistent in reporting the placement of the arterial pressure transducer [8, 14]. The Brain Trauma Foundation’s most recent guidelines recommend a CPP of 60–70 mm Hg in traumatic brain injury, with the arterial pressure transducer calibrated at the level of the right atrium [5]. However, it is not entirely clear at what level the transducer has been placed in the studies that these recommendations are based on. Moreover, care must also be taken to define “calibrated” so that it is not to be confused with the level at which the pressure transducer is “zeroed” or “calibrated” to air pressure, which is inconsequential, as this difference is negligible. We suggest a preferred terminology indicating where pressure transducers are leveled. In aggregate, inconsistency and underreporting of the transducer leveling point and the margins of error this causes have contributed to significant uncertainty regarding the interpretation of these guidelines.

To circumvent these sources of confusion and to increase patient safety, a dual-transducer approach from a single arterial line has been used at the Karolinska University Hospital. All patients receiving an ICP monitoring device will also have an invasive dual-transducer arterial pressure monitoring system as described below.

## The Dual-Transducer System

This dual-transducer system has been granted regulatory approval in Europe and is now available commercially. None of the authors have any financial interests concerning this product.

**Fig. 1:**
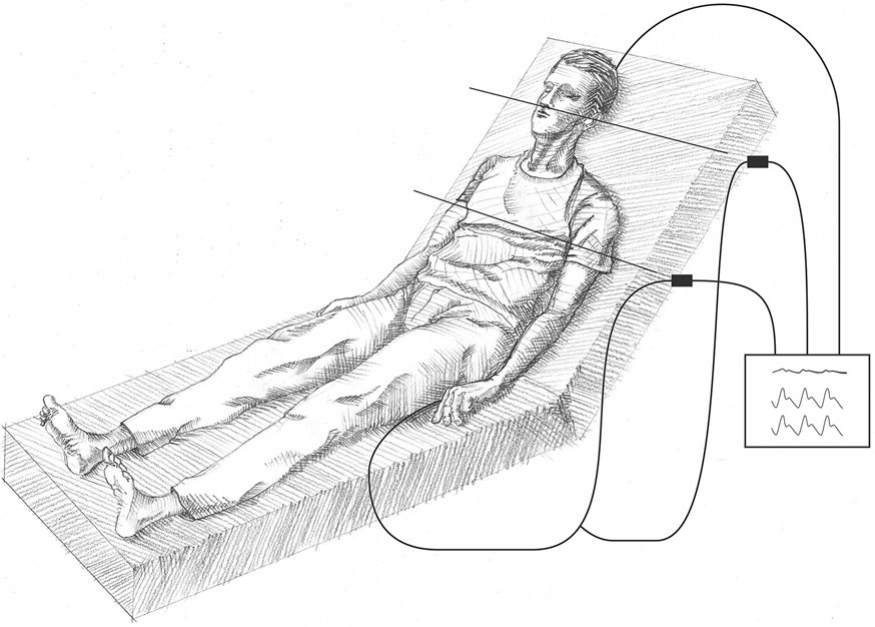
Illustration of the split dual-transducer system with two transducer systems connected to a single arterial line for simultaneous arterial blood pressure measurement from both the phlebostatic axis and tragus

**Fig. 2:**
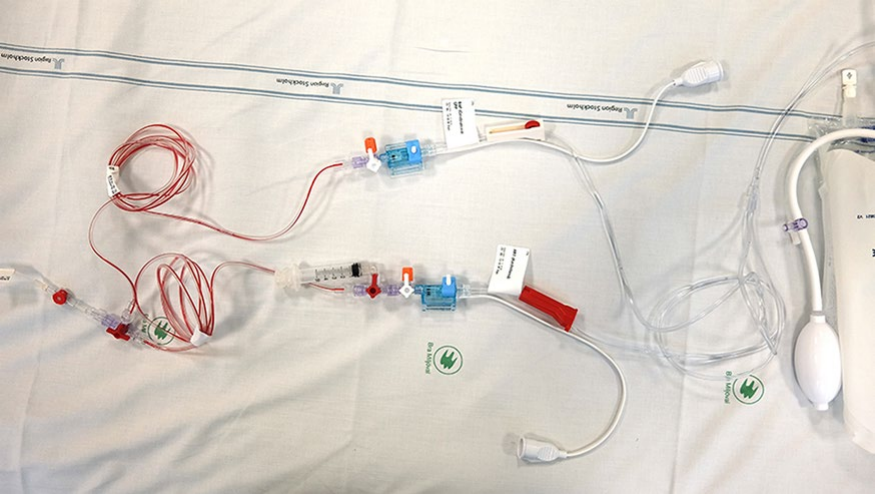
The dual-transducer kit. The transducers are name tagged and have roller stoppers of the corresponding color on the monitor (white for ABP_tragus_ and red for ABP_phlebo_). ABP_phlebo_ arterial blood pressure measured at the phlebostatic axis, ABP_tragus_ arterial blood pressure measured at the tragus

In the dual-transducer system, dual transducers are connected and receive pressures via a common arterial line (Figs. 1 and 2). A system like this must be constructed such that it does not affect compliance and dynamic response of the system [18]. One transducer is placed at the level of the right atrium, and the other is placed at the level of the cerebral pressure measurement, commonly at the level of the tragus. The CPP is calculated by subtracting the ICP from the MAP_tragus_ and is presented on the monitor in the color of the ICP and cerebral arterial blood pressures (ABP_tragus_) (commonly white). The systemic arterial pressure (ABP_phlebo_) values are traditionally displayed in red. Care must be taken that pressure transducers or calculations are not inverted. This is easily recognizable, as the MAP_phlebo_ is higher than the MAP_tragus_ (commonly by 11–13 mm Hg), and CPP is correctly seen as MAP_tragus_ − ICP. This system is used in our setting whenever a continuous ICP measure of any source is present.

From a practical nursing perspective, the common arterial line and each transducer are operated in the same way as with previous single systems, although it is periodically necessary for both transducer lines to be flushed. The transducers are color and name tagged (in our unit, ART [Arterial] for ABP_phlebo_ and BAP [brain arterial pressure] for ABP_tragus_) (Fig. 2), corresponding to colors on the screen. The ABP_phlebo_ and its signal are displayed in standardized red, with ABP_tragus_, ICP, and CPP displayed in white. A routine check process has been implemented in which staff at handovers or after transport ensure that the transducers have not been physically displaced and that CPP is correctly calculated from MAP_tragus_: “Check MAP_phlebo_ > MAP_tragus_ and CPP = MAP_tragus_ − ICP (all white) on the monitor.”

## Discussion

Based on observation from our 5-year clinical experience with more than 3,000 units used, we suggest that the dual-transducer system may now be of interest to the wider community. Ideally, the arterial pressure transducer will be placed at the level of the tragus for CPP measurement and at the level of the phlebostatic axis for systemic measurement. The dual-transducer system overcomes the inherent trade-off of single-transducer systems in situations in which both systemic and cerebral pressures are of interest. To work with pressure evaluations directly supplied from levels of the organs of interest leads to less ambiguity. It could also more readily provide clinicians with critical information without the need for estimation or manual calculation, thus reducing the risk of human errors and overtreatment and undertreatment.

It is important to note that we have considered the hydrostatic differences here. The actual morphology of the arterial pulse as it reaches the brain and interacts with the vasculature will be different than that in the radial artery; this is a complex nonlinear dynamics effect that does not have a solution to date (save for direct measurement by a catheter in the carotid). However, our understanding of the physiology of autoregulation/cerebral perfusion to date has been predicated on the quasi-static case, and that is what this device seeks to better quantify.

To date, there are no clinical studies evaluating the utility of the split dual-transducer system. Our experience is that using the unit is feasible and technically tractable in a high-resource environment. We are here suggesting a simple technical solution to a situation that has been readily identified and has historically hampered CPP recommendations. In short, discussions of adequate CPP ranges have been within the near range of measurement error and variation. The value of limiting this inconsistency would be expected to be beneficial. However, to formally evaluate it, future evaluation in a large multicenter setting will be required, investigating, among others, the effects on the usage of fluids and vasopressors as well as safety issues associated with implementation. Finally, our hope is that a wider adoption of a dual-transducer system could also catalyze the homogenization of CPP measurement and allow us to draw more generalizable conclusions from future research studies.

## References

[1] Lassen NA (1959). Cerebral blood flow and oxygen consumption in man. Physiol Rev.

[2] Bouma GJ, Muizelaar JP, Bandoh K, Marmarou A (1992). Blood pressure and intracranial pressure-volume dynamics in severe head injury: relationship with cerebral blood flow. J Neurosurg.

[3] Jünger EC, Newell DW, Grant GA (1997). Cerebral autoregulation following minor head injury. J Neurosurg.

[4] Sahuquillo J, Munar F, Baguena M, Poca MA, Pedraza S, Rodríguez-Baeza A (1998). Evaluation of cerebrovascular CO_2_-reactivity and autoregulation in patients with post-traumatic diffuse brain swelling (diffuse injury III). Acta Neurochir Suppl.

[5] Carney N, Totten AM, O'Reilly C, et al. Guidelines for the management of severe traumatic brain injury. 4th ed. Palo Alto (CA): Brain Trauma Foundation; 2016.

[6] McNett MM, Bader MK, Livesay S (2018). A national trial on differences in cerebral perfusion pressure values by measurement location. Neurocrit Care.

[7] Kosty JA, Leroux PD, Levine J (2013). A comparison of clinical and research practices in measuring cerebral perfusion pressure: a literature review and practitioner survey. Anesth Analg.

[8] Jones HA (2009). Arterial transducer placement and cerebral perfusion pressure monitoring: a discussion. Nurs Crit Care.

[9] Olson DM, Batjer HH, Abdulkadir K, Hall CE (2014). Measuring and monitoring ICP in neurocritical care: results from a national practice survey. Neurocrit Care.

[10] McCann UG, Schiller HJ, Carney DE (2001). Invasive arterial BP monitoring in trauma and critical care. Chest.

[11] McNett M, Livesay S, Yeager S (2018). The impact of head-of-bed positioning and transducer location on cerebral perfusion pressure measurement. J Neurosci Nurs.

[12] Gardner RM (1990). Direct arterial pressure monitoring. Curr Anaesth Crit Care.

[13] Lamontagne F, Richards-Belle A, Thomas K (2020). Effect of reduced exposure to vasopressors on 90-day mortality in older critically ill patients with vasodilatory hypotension: a randomized clinical trial. JAMA.

[14] Rao V, Klepstad P, Losvik OK, Solheim O (2013). Confusion with cerebral perfusion pressure in a literature review of current guidelines and survey of clinical practice. Scand J Trauma Resusc Emerg Med..

[15] Lele AV, Wilson D, Chalise P, Nazzaro J, Krishnamoorthy V, Vavilala MS (2018). Differences in blood pressure by measurement technique in neurocritically ill patients: a technological assessment. J Clin Neurosci.

[16] Wiegers EJA, Lingsma HF, Huijben JA (2021). Fluid balance and outcome in critically ill patients with traumatic brain injury (CENTER-TBI and OzENTER-TBI): a prospective, multicentre, comparative effectiveness study. Lancet Neurol.

[17] Siaron KB, Cortes MX, Stutzman SE, Venkatachalam A, Ahmed KM, Olson DWM (2020). Blood pressure measurements are site dependent in a cohort of patients with neurological illness. Sci Rep.

[18] Ercole A (2006). Attenuation in invasive blood pressure measurement systems. Br J Anaesth.

